# Strategies for the implementation of the living guidelines for cochlear implantation in adults

**DOI:** 10.3389/fpubh.2023.1272437

**Published:** 2023-12-15

**Authors:** Ángel Ramos-Macías, Leo De Raeve, Meredith Holcomb, Ella Connor, Aiya Taylor, Irene Deltetto, Colman Taylor

**Affiliations:** ^1^Department of Otolaryngology, School of Medicine, University of Las Palmas, Las Palmas, Spain; ^2^Independent Information and Research Centre on Cochlear Implants (ONICI), European Association of Cochlear Implant Users (EURO-CIU) and Cochlear Implant International Community of Action (CIICA), Zonhoven, Belgium; ^3^Department of Otolaryngology, University of Miami Miller, School of Medicine, Miami, FL, United States; ^4^HTANALYSTS, Sydney, NSW, Australia; ^5^Critical Care, The George Institute for Global Health, Sydney, NSW, Australia

**Keywords:** living guidelines, cochlear implantation, hearing loss, guideline implementation strategies, guideline implementation tools

## Abstract

**Introduction:**

Clinical guidelines for cochlear implants (CI) exist in several countries, however, they lack consistency and often do not encompass the full user journey. This study aims to explore the barriers and facilitators for implementing global Living Guidelines for cochlear implantation in adults with severe, profound or moderate sloping to profound sensorineural hearing loss (SPSNHL) as well as identify guideline implementation (GI) tools that may support uptake.

**Methods:**

A convenience sample of Task Force members were recruited for semi-structured interviews. Interview transcripts were thematically analysed to group country-specific barriers, facilitators and GI tools into three levels: health care provider (HCP), consumer and structural. Once identified, barriers and facilitators were classified into four themes related to awareness, economic, guideline or other.

**Results:**

Interviews were conducted with 38 Task Force members, representing 20 countries. Lack of CI and hearing loss awareness was a major barrier at the HCP (85% of countries), consumer (80%) and structural (20%) levels. Economic and guideline barriers followed at the HCP (35%; 25%), consumer (45%; 0%) and structural (55%; 30%) levels, respectively. Facilitators focused on raising awareness of hearing loss and CIs as well as guideline related initiates at the HCP (80%; 70%), consumer (70%; 10%) and structural (25%; 70%) levels. GI tools including education, economic evaluations, quick reference resources and social media can help improve awareness and uptake.

**Conclusion:**

Awareness is the primary barrier to implementing Living Guidelines globally for adults with SPSNHL. Endorsement from key professional bodies and using the best available evidence can enhance uptake.

## 1 Introduction

Hearing loss is one of the most prevalent and undertreated disabilities worldwide (1). Despite cochlear implants (CIs) being a safe and effective treatment option for adults with severe, profound or moderate sloping to profound sensorineural hearing loss (SPSNHL) (2), it is estimated that at best only one in 20 adults who would benefit from a CI have received one (2–4).

In 2020, an international group of clinical hearing loss experts highlighted that while cochlear implantation should be standard of care in adults with SPSNHL, it is underutilized as a treatment option, suggesting there is an urgent need to address the lack of globally consistent CI guidelines (2, 5).

Currently, some countries have detailed guidelines with varying levels of uptake for candidacy requirements [United states (US)] (2, 6), pre-operative evaluation (Australia, Germany, France, Spain, US) (6–12), CI surgery (Australia, England, Germany, France, Spain, US) (6–10, 13–16), and post-operative care (Australia, Germany, US) (6–8, 11, 12). However, holistic international guidelines on adult CI treatment are limited (2).

Therefore, in 2022, an independent global CI Task Force was established to guide the development of new global ‘Living’ Guidelines for CI in adults with SPSNHL, with the aim to improve the standard of care for these individuals and provide guidance on treatment options. The concept of living guidelines is a new approach to driving evidence-based practice, and will ensure that recommendations within the Living Guidelines are always underpinned by a rigorous evidence base and remain relevant within a continuously evolving field of research (17).

Once published in early 2023, the Living Guidelines will be implemented in multiple countries. Although, numerous studies emphasize challenges with translating recommendations from clinical guidelines into practice, suggesting they require adaptation and adoption at micro- (e.g., patients and clinicians), meso- (e.g., the healthcare system) or macro- (e.g., government and regulation) environments (18).

Thus, the primary objective of this study was to explore the barriers and facilitators for successful implementation of the Living Guidelines, and to understand what guideline implementation (GI) tools will be required to support uptake.

## 2 Methods

### 2.1 Study design

The global CI Task Force comprised of 52 Task Force members, including 30 Ear, Nose and Throat Specialists (ENTs), 16 audiologists, 4 cochlear impact users and 2 hearing specialists (one ENT and one audiologist) who are also users. Three co-chairs were appointed to lead the CI Task Force, who represented each stakeholder group at a leadership level (one ENT, one audiologist and one user). The members of the CI Task Force were selected and recommended by peers to ensure a balance of global geographical location, gender and expertise.

All Task Force members were invited to participate in semi-structured interviews. Task Force members were contacted via email in September 2022 and one-on-one interviews were conducted remotely in October 2022. Participants provided both written and verbal consent prior to commencing their interview.

### 2.2 Sampling and recruitment

The study recruited a convenience sample of members from the Task Force. Overall, 38 Task Force members from 20 countries consented to be interviewed and this sample size was expected to be sufficient to meet the project objectives. The overall response rate was 73%, ranging from 67% (Latin America) to 80% (Asia-Pacific) (please see Supplementary Table 1).

### 2.3 Data collection

Interviews were conducted remotely by two investigators (AT, EC) who had minimal prior experience conducting research on hearing loss and CI care. Thus, researchers had minimal existing biases, which allowed themes to emerge naturally. Interviews lasted approximately 1 h and were audio-recorded and transcribed verbatim.

### 2.4 Data analysis

Three investigators (ID, AT, EC) categorized barriers and facilitators into three levels: health care provider (HCP) (audiologists, otolaryngologists), consumer (CI users, families) and structural (the healthcare system). This methodology was considered appropriate, as these categories related to the interview questions (please see Supplementary Appendix A). Following this analysis, two researchers (AT, EC) further grouped themes into four categories including awareness (related to the knowledge of hearing loss and CIs in each county), economic (related to the economic environment, laws and regulations), guideline specific (related to the actual implementation of the Living Guidelines) and other (additional barriers and facilitators).

To analyse the results, the proportion of countries (*N* = 20) that reported an individual barrier and facilitator within each of these pre-defined levels and categories were calculated. When multiple participants from the same country reported a barrier or facilitator, this was considered to be one vote for the respective barrier or facilitator category.

GI tools were also grouped according to the HCP, consumer and structural levels, to facilitate these being mapped to the barriers and facilitators identified. The results were presented according to the proportion of countries (*N* = 20) that recommended each category of GI tool.

### 2.5 Ethics

Ethics approval was granted by the Bellberry Human Research Ethics Committee (No. 2022 06656).

## 3 Results

### 3.1 Barriers and facilitators

The proportion of countries that reported barriers and facilitators in each category is summarized in Table 1. Overall, a total of 28 barriers and 28 facilitators were identified across all levels, presented in Tables 2, 3, respectively.

**Table 1:** Summary of themes (barriers and facilitators) identified by countries at health care provider, consumer and structural levels.

Level	Category	Barriers		Facilitators	
		*n*	*n* (*n*/*N*) *N* = 20	*n*	*n* (*n*/*N*) *N* = 20
Health care provider	Awareness	17	85%	16	80%
	Economic	7	35%	0	0%
	Guideline specific	5	25%	14	70%
	Other	3	15%	4	20%
Consumer	Awareness	16	80%	14	70%
	Economic	9	45%	0	0%
	Guideline specific	0	0%	2	10%
	Other	0	0%	0	0%
Structural	Awareness	4	20%	5	25%
	Economic	11	55%	2	10%
	Guideline specific	6	30%	14	70%
	Other	11	55%	0	0%

*n*, total number of times a theme (barrier and facilitator) was raised by individual countries; *N*, total number of countries.

One country was equal to one vote per category (barriers and facilitators).

**Table 2:** Themes (barriers) reported by countries influencing implementation of the Living Guidelines at health care provider, consumer and structural levels.

Level	Category	Theme (barrier)	*n* (*n*/*N*) *N* = 20
Consumer	Awareness	Some consumers are unaware of the benefits of CI and/or that it is a treatment option.	14 (70%)
Health care provider	Awareness	GPs lack knowledge about hearing health.	8 (40%)
Structural	Other	Laws and requirements (e.g., public vs. private, different states).	8 (40%)
Health care provider	Awareness	There is a disconnect or lack of communication between clinical audiologists, CI teams and users.	7 (35%)
Health care provider	Awareness	GPs are overworked and may not use another guideline.	7 (35%)
Consumer	Awareness	Consumers do not recognize the broader impacts of hearing loss.	7 (35%)
Structural	Economic	Limited resources and high demand.	7 (35%)
Health care provider	Awareness	Audiologists are not aware of CI eligibility criteria and its benefits.	6 (30%)
Health care provider	Awareness	Audiologists are reluctant and unsure how to refer CI patients.	6 (30%)
Consumer	Awareness	Stigma and fear of acceptance associated with CIs and hearing loss.	6 (30%)
Health care provider	Awareness	The wider MDT (e.g., geriatricians) are not involved in hearing care.	5 (25%)
Health care provider	Awareness	Hearing loss is not viewed as a chronic disease and thus not taken ‘seriously’ by all clinicians.	4 (20%)
Structural	Awareness	Hearing loss will not be a critical area of health focus in the near future.	4 (20%)
Health care provider	Awareness	Otolaryngologists do not understand the value of CI.	3 (15%)
Consumer	Economic	If recommendations cost the consumer or healthcare system, they are less likely to be implemented.	3 (15%)
Health care provider	Economic	Audiologists are focused on hearing aid sales and often do not refer patients to specialists.	7 (35%)
Consumer	Economic	Limited resources and high demand.	7 (35%)
Health care provider	Guideline specific	Lack of willingness to change or follow guidelines.	5 (25%)
Structural	Guideline specific	Competing guidelines with different recommendations may cause confusion.	4 (20%)
Health care provider	Other	Knowledge gap between older and newer audiologists.	3 (15%)
Structural	Economic	If recommendations cost the consumer or healthcare system, they are less likely to be implemented.	3 (15%)
Structural	Other	Disconnect between CI treatment and rehabilitation.	3 (15%)
Consumer	Awareness	Social barrier – elderly do not think they need any hearing care and save more for children.	2 (10%)
Structural	Economic	Audiologists are not adequately reimbursed for the time required to carry out CI assessments.	2 (10%)
Structural	Economic	Few state funded CI programs and most do not cover rehabilitation and aftercare.	2 (10%)
Structural	Guideline specific	Strict CI eligibility criteria.	2 (10%)
Consumer	Awareness	Consumers are confused as to what is covered by CI insurance.	1 (5%)
Structural	Guideline specific	Under-representation from the user perspective during the guideline development process.	1 (5%)

CI, cochlear implant; MDT, multi-disciplinary team; *n*, total number of countries that raised the barrier; *N*, total number of countries interviewed.

One country was equal to one vote per theme (barrier).

**Table 3:** Themes (facilitators) reported by countries influencing implementation of the Living Guidelines at health care provider, consumer and structural levels.

Level	Category	Theme (facilitator)	*n* (*n*/*N*) *N* = 20
Health care provider	Guideline specific	Have endorsement from key formal and informal stakeholders.	14 (70%)
Health care provider	Awareness	Improve awareness of the Living Guidelines across the MDT.	9 (45%)
Consumer	Awareness	Educate patients and families on the benefits of CI.	9 (45%)
Structural	Guideline specific	Ensure the Living Guideline recommendations are applicable to all key stakeholders.	9 (45%)
Health care provider	Awareness	Provide clarity on where the new Living Guidelines fit within existing protocols and guidelines.	8 (40%)
Health care provider	Awareness	Improve awareness of the Living Guidelines among primary health care providers.	7 (35%)
Consumer	Awareness	Involve patient advocacy organizations.	7 (35%)
Consumer	Awareness	Improve awareness about the broader impact of hearing loss.	7 (35%)
Structural	Guideline specific	Increase rehabilitation support post-implantation.	7 (35%)
Structural	Guideline specific	Ensure Living Guideline recommendations or GI tools are localized to current clinical practice.	6 (30%)
Health care provider	Awareness	Target and approach providers who are less convinced of CI.	5 (25%)
Health care provider	Guideline specific	Develop the Living Guidelines using the best available evidence.	5 (25%)
Structural	Guideline specific	Ensure the Living Guidelines have wide accessibility.	5 (25%)
Health care provider	Awareness	Motivation for the new Living Guideline needs to be explained and contextualized.	4 (20%)
Health care provider	Guideline specific	Ensure there the link is clear between current guidelines and the Living Guidelines.	4 (20%)
Health care provider	Other	Incorporate the Living Guideline recommendations within existing education curriculums.	4 (20%)
Health care provider	Guideline specific	Have the Living Guidelines published in medical journals.	3 (15%)
Health care provider	Guideline specific	Information and objectives need to be clear and simply presented to be understood.	3 (15%)
Consumer	Awareness	Reduce stigma associated with hearing loss and CI.	3 (15%)
Structural	Awareness	Education for government bodies.	3 (15%)
Structural	Awareness	Need support from audiology clinics.	3 (15%)
Structural	Guideline specific	Enforce requirements to screen and refer patients.	3 (15%)
Structural	Guideline specific	Enforce requirements to screen and refer patients.	3 (15%)
Consumer	Guideline specific	Ensure Living Guideline recommendations are relatable to the patient.	2 (10%)
Structural	Guideline specific	Government relationships.	2 (10%)
Structural	Economic	Identify funding from other professional organizations to fund the Living Guidelines.	1 (5%)
Structural	Economic	Align with payer guidelines.	1 (5%)
Structural	Guideline specific	Have CI companies involved in the guideline development process.	1 (5%)
Structural	Guideline specific	Follow formal country-specific guideline development requirements.	1 (5%)

CI, cochlear implant; MDT, multi-disciplinary team; *n*, total number of countries that raised the facilitator; *N*, total number of countries interviewed.

One country was equal to one vote per theme (facilitator).

#### 3.1.1 Health care provider

Awareness was identified as the largest HCP barrier (85%) that could impact the successful adoption of the Living Guidelines (Table 1). Frequently reported themes included a lack of general practitioner (GP) (40%) and audiologist (30%) knowledge about hearing health or the value of CIs, and a disconnect or lack of communication between primary HCPs, audiologists and otolaryngologists (35%) (Table 2). Additionally, the largest economic barrier was a lack of referrals from audiologists (35%), and the largest guideline specific barrier was a lack of willingness to change practice or follow guidelines (25%) (Table 2).

*“Awareness is key. I've been doing this for years and every day I'm surprised by how many GPs and audiologists don't know about cochlear implants (CIs).” – Task Force member, Canada*

*“I think one of the biggest issues is that lots of people do not realise that they do better with a CI than they would do with hearing aids.” – Task Force member, Australia*

Most countries (80%) reported improving awareness as the most important facilitator for HCPs (Table 1). Specifically, improving awareness of the Living Guidelines across the multi-disciplinary team (MDT) (45%) and primary HCPs (35%) (Table 3). Furthermore, providing clarification on how the Living Guidelines align with existing national protocols or guidelines (40%) and having them endorsed by key professional bodies (70%) were the common guideline specific facilitators reported (Table 3). Others included using the best available evidence (25%) to develop recommendations and ensuring there is a clear link between current guidelines and the Living Guidelines (20%) (Table 3).

*“A global standard for treatment for hearing loss would go a long way in helping patients.” – Task Force member, United States of America*

#### 3.1.2 Consumer

Awareness was identified as a major consumer barrier (80%) (Table 1). This included a lack of awareness of the potential benefits of CIs (70%) and hearing loss consequences (35%), as well as the associated stigma and fear of acceptance (30%) (Table 2). Approximately half of the participating countries (45%) (Table 1) reported economic barriers associated with the cost of CIs (40%) and travel required for care (30%) (Table 2).

Improving consumer awareness was seen as a critical facilitator in most countries (70%) (Table 1). Specific awareness facilitators related to educating (45%) and improving awareness (35%) about the broader impacts of hearing loss and the value of CIs (Table 3). Furthermore, involving patient advocacy organizations during implementation was also considered essential (35%) (Table 3).

*“The biggest thing is to make sure we (users) have access to the information” – Task Force member, Australia*

#### 3.1.3 Structural

Economic (55%) and other barriers (55%) were the most frequently reported for the structural level (Table 1). For instance, having limited resources to meet patient demand (35%) and the laws and regulations across healthcare systems (40%) (Table 2).

*“The economics of hearing care do not incentivise providers to offer cochlear implants because every time a private practice audiologist or hearing instrument specialist offers an implant, they're offering to give away a patient.” – Task Force member, United States of America*

Regarding structural facilitators, most suggestions related to the content of the Living Guidelines (70%) (Table 1). This included ensuring the recommendations will be applicable to all key stakeholders (45%) and increasing rehabilitation support services (35%) (Table 3).

### 3.2 Guideline implementation tools

The proportion of countries that identified GI tools for each level is summarized in Table 4. Overall, 42 different GI tools were recommended to support implementation and uptake of the Living Guidelines (see Supplementary Appendix B).

**Table 4:** Summary of themes (GI tools) identified by countries at health care provider, consumer and structural level.

Level	Category	*n* (*n*/*N*) *N* = 20	Description
Health care provider	Education	18 (90%)	Print, electronic or in-person information about the Living Guidelines, CIs and/or hearing loss targeting audiologists, otolaryngologists, primary health care professionals or hearing aid dispensers.
	Advertisement and support via key professional bodies	13 (65%)	Endorsement, support and distribution of information about the Living Guidelines via key formal and informal stakeholders.
	Quick reference resources	12 (60%)	Resources such as discussion guides, PowerPoint slides, referral guides, charts, templates, screening tools, patient tracking and targeted guideline summaries that can be quickly and easily used by clinicians to support patient care.
	Local leaders	3 (15%)	Leaders or local authorities to promote the Living Guidelines amongst key stakeholders such as primary HCPs (e.g., GPs), audiologists, otolaryngologists and HCPs outside of the hearing health setting.
Consumer	Education	13 (65%)	Print, electronic or in-person information about the Living Guidelines, CIs and/or hearing loss targeting users or families.
	Quick reference resources	12 (60%)	Resources such as brochures, posters, clinical pathways, self-testing tools, shared decision-making tools, virtual programs and a summary of the Living Guidelines that can be easily accessed and used by the community to guide hearing loss care.
	Social media	12 (60%)	User stories, campaigns and/or influencers promoting and educating the community about the Living Guidelines, CIs and hearing loss via non-biased and non-commercial platforms (Facebook, YouTube, LinkedIn, Instagram etc) or apps.
	Local leaders	4 (20%)	Leaders or local authorities to promote the Living Guidelines among consumers, families and patient advocacy organizations.
	Leverage AI advantages of CI	2 (10%)	Devices that enhance communication between adults with hearing loss, such as speakers and mobile phones, to enhance CI attractiveness.
Structural	Audit tools	6 (30%)	Methods to support the evaluation of guideline-compliant practice before and after implementation.
	Economic evaluation	6 (30%)	Economic evaluation of the cost savings stemming from implementation and uptake of the Living Guidelines.
	Social impact analysis	5 (25%)	Social impact analysis of the broader impacts of untreated hearing loss.
	Publication of the Living Guidelines	3 (15%)	Public publication of the Living Guidelines.
	Trial of the Living Guidelines	1 (5%)	Trial of the Living Guidelines in a local context prior to final completion of recommendations.

AI, artificial intelligence; CI, cochlear implant; GPs, general practitioners; HCPs, health care providers; *n*, total number of countries that raised a GI tool; *N*, total number of countries interviewed.

One country is equal to one vote per type of GI tool.

#### 3.2.1 Health care provider

Eighteen GI tools were recommended to support HCPs (Appendix B). Overall, education (90%), advertisement or support by key professional bodies (65%) and quick reference resources (60%) were the most frequently reported (Table 4).

*“It is important to get endorsement from all of the professional groups who already create these kinds of document.” – Task Force member, Sweden*

#### 3.2.2 Consumer

Nineteen GI tools were recommended to support consumers (Appendix B). Education (65%), social media (60%) and quick reference resources (60%) were the most commonly nominated (Table 4).

*“We need to build "pro-tools”, which are built from the ground up, built from the recipients, from patients, from people. This is going to be the most important thing. – Task Force member, United States of America*

#### 3.2.3 Structural

Five GI tools were recommended to support implementation at the structural level (Appendix B). An audit tool (30%), economic evaluation (20%) and social impact analysis (25%) were the most frequently suggested (Table 4).

*“There must be an audit or evaluation tool. It is important to monitor the impact of these guidelines - take up rates, influence, impact”– Task Force member, India*

## 4 Discussion

In this convenience sample of representatives from the CI Task Force, we identified several barriers, facilitators and GI tools related to the successful implementation of the Living Guidelines globally. Awareness of CIs and the impact of hearing loss were identified as major barriers across the HCP, consumer and structural levels. This was followed by economic barriers, such as out-of-pocket costs, and barriers related to the Living Guidelines, such as under-representation of CI users in guideline development. Facilitators mainly focused on increasing awareness of CIs and the impact of hearing loss as well as guideline related initiatives, such as gaining endorsement from key professional bodies. Finally, education, economic evaluations and ‘wrap-around tools’ including quick reference resources and social media campaigns were nominated to facilitate uptake.

To ensure this is successful, our findings demonstrate that the lack of GP, audiologist and consumer awareness must be addressed. These results are consistent with implementation research previously conducted in other healthcare areas that also identified a lack of HCP knowledge and time as significant barriers (18–21). For this to be addressed, CI specialists must receive appropriate training and education on the Living Guideline recommendations to ensure key stakeholders in their countries understand how they align with current clinical practice. Furthermore, gaining endorsement from these stakeholders will be a critical step to ensuring rapid uptake.

Additionally, educating non-CI specialists — including GPs, audiologists not CI trained, otolaryngologists who do not perform CI surgeries and hearing aid dispensers — about hearing health and CI benefits is crucial. These clinicians are commonly the first point of contact for potential CI candidates. Thus, enhancing their knowledge is important for improving CI referrals and patient management. This can be supported with quick reference resources, such as a lay summary of the Living Guideline recommendations. Improving knowledge, motivation and sense of responsibility among GPs have previously been identified in literature as influential facilitators for greater compliance of guidelines (22).

Increasing primary HCPs awareness will ultimately address knowledge concerns at a consumer level, by improving access to information. This may reduce the stigma related to CIs and empower adults with hearing loss to be involved in their treatment decisions, especially if supported by social media and quick reference resources. Specifically, a tool able to drive demand for a hearing assessment or intervention should be developed, using lay language to ensure equity and inclusion. Our results encourage the development of such resources to improve universal awareness about the broader consequences of untreated hearing loss such as depression, dementia, diabetes or heart disease (2, 23). Globally, it was suggested that this is not well understood and is thus a key facilitator to ensure successful adoption.

Our findings address several gaps in the literature regarding implementation strategies, as majority of existing GI tools target clinicians (52%) rather than patients (24%), implementation (14%) or evaluation (10%) (24). Furthermore, the importance of evaluating the uptake of guidelines is consistently recognized in literature (24, 25), but performing economic and social impact analyses are newly identified tools to our knowledge.

### 4.1 Strengths and limitations

Worldwide, this research is the first to examine living guideline implementation strategies for adults with SPSNHL, which is a key strength. Additionally, the inclusion of expert and influential audiologists, otolaryngologists and CI users, who represented a diverse range of developed and developing countries, may enhance the transferability of research findings.

However, some limitations may impact the interpretation and application. First, recruitment was restricted to the Task Force, which included a relatively small proportion of CI users, and excluded GPs and HCPs outside of the hearing health setting. Therefore, it is possible that important barriers, facilitators or GI tools may have been overlooked. Second, all findings are based on expert opinion from top leaders in the field and are not supported by evidence-based data. Third, some of the structural barriers identified, such as country-specific laws or CI cost and reimbursement, will be difficult to address without significant reform. Fourth, several Task Force members from African countries were unavailable to participate, and therefore this study was not representative of the global population.

Furthermore, it is important to acknowledge that inherent biases to specialist observations may be present in the results, as the reasons provided and presumed countermeasures are based on consensus, rather than scientific exploration of root causes. However, there is value in using these assessments as a starting point, recognizing that successful adoption of future living guidelines will need to be monitored, and plans adjusted based on observation and data.

## 5 Conclusion

This study emphasizes the importance of utilizing GI implementation tools to ensure the successful implementation and widespread adoption of the Living Guidelines, based on expert opinion. In particular, findings indicate that addressing the limited awareness of hearing loss and CIs worldwide, as well as economic barriers such as referral channels and out-of-pocket costs, are key to achieving this goal. Although, many practical steps can be undertaken to assist implementation including endorsement by professional bodies and ensuring the recommendations adhere to the best available evidence. Supporting these with economic evaluations, quick reference resources and social media campaigns could help facilitate uptake of the Living Guidelines in individual countries.

## Data availability statement

The raw data supporting the conclusions of this article will be made available by the authors, without undue reservation.

## Ethics statement

The studies involving humans were approved by Bellberry Human Research Ethics Committee. The studies were conducted in accordance with the local legislation and institutional requirements. The participants provided their written informed consent to participate in this study.

## Author contributions

ÁR-M: Validation, Writing – review & editing. LR: Validation, Writing – review & editing. MH: Validation, Writing – review & editing. EC: —. AT: Writing – review & editing. ID: Writing – review & editing. CT: Writing – review & editing.
