# Supplementary material for: Strategies for the implementation of the living guidelines for cochlear implantation in adults
Source: Front Public Health. 2023 Dec 15;11:1272437. doi: 10.3389/fpubh.2023.1272437 (PMC10757841; doi:10.3389/fpubh.2023.1272437)
Supplement: Supplementary file 1 [file Data_Sheet_1.pdf]

Supplementary Material

Table 1 Geography and response rate of Task Force members

**Region**	**N**	**n**	**Response rate (n/N)**	**Country (n)**
APAC	15	12	80%	Australia (7), India (1), Japan, (1), New Zealand (1), Singapore (1), South Korea (1)
CAM	17	12	71%	Canada (1), United States (11)
EMEA	16	12	75%	Belgium (1), England (2), Germany (2), Israel (1), Italy (1), Poland (1), Scotland (1), Slovenia (1), Spain (1), Sweden (1)
LATAM	3	2	67%	Argentina (1), Colombia (1)
TOTAL	52	38	73%	20 Countries

Abbreviations: APAC, Asia-Pacific; CAM, Central American Region; EMEA, Europe, Middle East and Africa; LATAM, Latin America

Appendix A

Interview questions (implementation)

1. Are you aware of any specific guideline requirements that must be met in your country?

2. Are you aware of any formal or informal stakeholders in your country that should endorse the Living Guidelines to ensure they are relevant?

3. From your perspective, what are the barriers to successful implementation of the Living Guidelines in your country for the following environments? (Micro: patients and clinicians; meso: the healthcare system; macro: government, regulation and economic).

4. From your perspective, what are the facilitators to successful implementation of the Living Guidelines in your country for the following environments? (Micro: patients and clinicians; meso: healthcare system; macro: government, regulation and economic).

Interview questions (uptake)

1. Are you aware of any existing practical tools relevant to the care of adults with hearing loss that could be adapted to support the Living Guidelines in your country?

2. From your experience, are there any guideline implementation tools that could be developed and used alongside the Living Guidelines in your country?

Appendix B

Table 2 Themes (GI tools) influencing implementation of the Living Guidelines at health care provider, consumer and structural levels

Level	Category	Theme (GI tool)	n (n/N) N=20
Health care provider	Education	Targeted at audiologists	7 (35%)
		Targeted at otolaryngologists	6 (30%)
		Targeted at primary health care providers	3 (15%)
		Targeted at all health care providers (including the MDT)	7 (35%)
		Targeted at hearing aid dispensers	1 (5%)
		Conferences with all key stakeholders	5 (25%)
	Advertisement and support via key professional bodies	--	13 (65%)
	Quick reference resources	60/60 posters	2 (10%)
		Posters	2 (10%)
		Flow charts	2 (10%)
		Referral guides	1 (5%)
		Discussion guides	2 (10%)
		Targeted fact sheets	1 (5%)
		Clear care pathways	2 (10%)
		Screening tools	1 (5%)
		Electronic patient tracking tools	1 (5%)
		Lay summaries for various stakeholders	3 (15%)
	Local leaders or champions	--	3 (15%)

Consumer	Education	Targeted at patients	12 (60%)
		Targeted and families and communities	8 (40%)
		Events/conferences with patient organisations	3 (15%)
	Quick reference resources	Brochures	1 (5%)
		Information in waiting rooms	3 (15%)
		Booklets to guide and document care	1 (5%)
		Lay summaries/guidelines adapted for users and families	4 (25%)
		Rehabilitation support or virtual rehabilitation	2 (10%)
		Self-test hearing screening or incentives	4 (25%)
		Shared decision-making tools	2 (10%)
	Leverage AI advantages of CIs	--	3 (15%)
	Local leaders or champions	--	4 (20%)
	Media (tv, radio, advertising, print)	--	3 (15%)
	Social media	Campaigns	5 (25%)
		Via a non-commercial or non-bias sources	2 (10%)
		Platforms (Facebook, YouTube, LinkedIn etc)	3 (15%)
		Influencers	1 (5%)
		User stories	5 (25%)
		Apps	4 (20%)
Structural	Audit tools	--	6 (30%)

	Economic evaluation	--	6 (30%)
	Trial of the Living Guidelines	--	1 (5%)
	Social impact analysis	--	5 (25%)
	Publication of the Living Guidelines	--	3 (15%)

Abbreviations: AI, artificial intelligence; CIs, cochlear implants; MDT, multi-disciplinary teams; n, total number of countries that raised the GI tool; N, total number of countries interviewed
Note: One country was equal to one vote per theme (GI tools
